# Three Times Recurrent Takotsubo Syndrome: An Educational Presentation

**DOI:** 10.21470/1678-9741-2022-0951

**Published:** 2022

**Authors:** Pedro Henrique R. e Silva, Jairo Rosa e S. Junior, Paulo Roberto B. Evora

**Affiliations:** 1 Faculdade de Ciências Médicas, Universidade Estadual de Campinas (UNICAMP), Campinas, São Paulo, Brazil.; 2 Faculdade de Medicina de Ribeirão Preto, Universidade de São Paulo (FMRP-USP), Ribeirão Preto, São Paulo, Brasil.; 3 Department of Surgery and Anatomy, Faculdade de Medicina de Ribeirão Preto, Universidade de São Paulo (FMRP-USP), Ribeirão Preto, São Paulo, Brazil.

**Table:** 

Abbreviations, Acronyms & Symbols	
ACE	= Angiotensin-converting enzyme
ACS	= Acute coronary syndrome
CATH	= Cineangiocoronariography
ECG	= Electrocardiogram
Echo	= Echocardiogram
ER	= Emergency room
LV	= Left ventricular
TTS	= Takotsubo syndrome

## INTRODUCTION

Female patient, 74 years old, with a history of hypothyroidism, diabetes mellitus type II, arterial hypertension (with mild ventricular hypertrophy), and dyslipidemia. She also had a chronic anxiety condition, with no psychotherapeutic accompaniment.

**Fig. 1:**
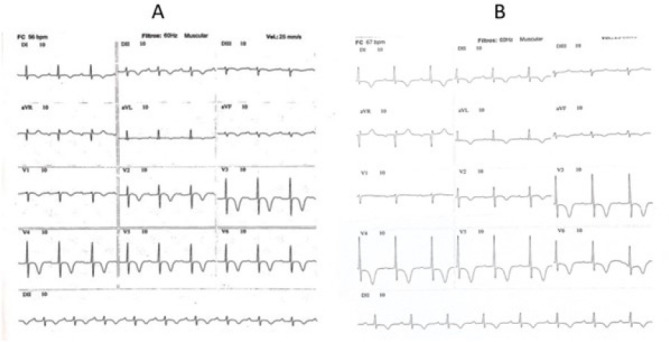
A) Electrocardiogram (ECG) from 2013. B) ECG from 2015. In A) and B) we can see peaked, inverted, and symmetrical T waves in V2, V3, V4, and V5.

**Fig. 2:**
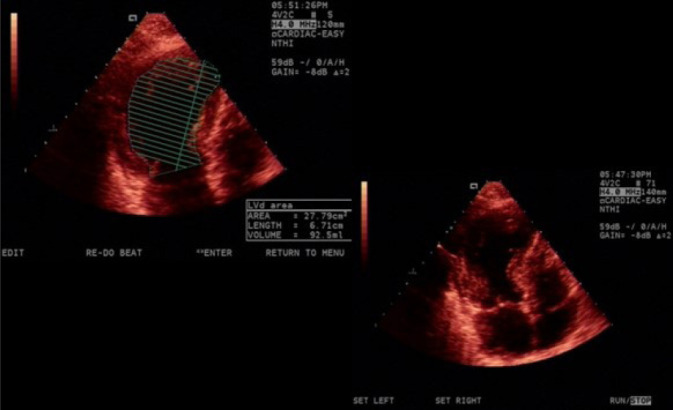
Echocardiogram (diastole and systole) from February 6, 2013 showing the typical Takotsubo left ventricular abnormality.

**Fig. 3:**
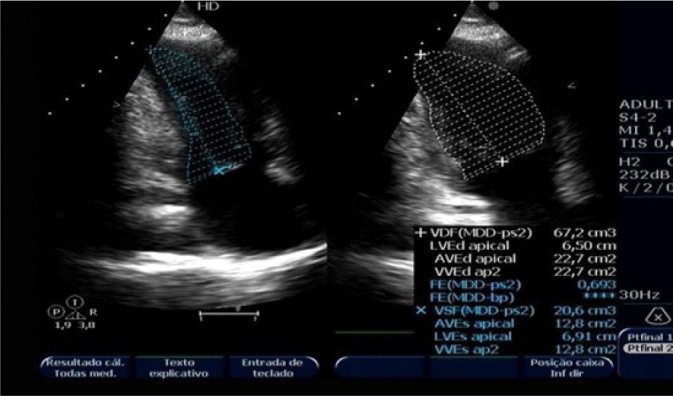
Echocardiogram (diastole and systole) from April 3, 2015 showing left ventricular abnormality, with the typical Takotsubo format.

**Fig. 4:**
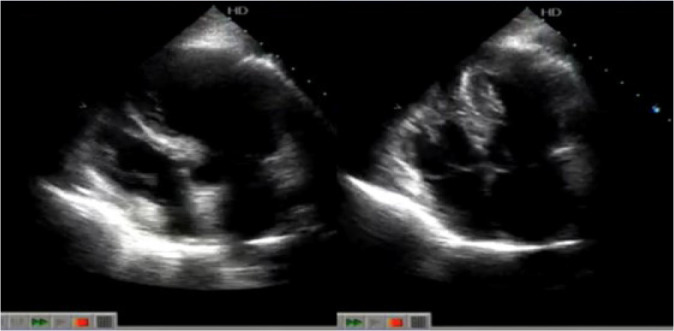
Echocardiogram (diastole and systole) from November 25, 2018 showing abnormality in the left ventricle, with the typical Takotsubo format.

**Fig. 5:**
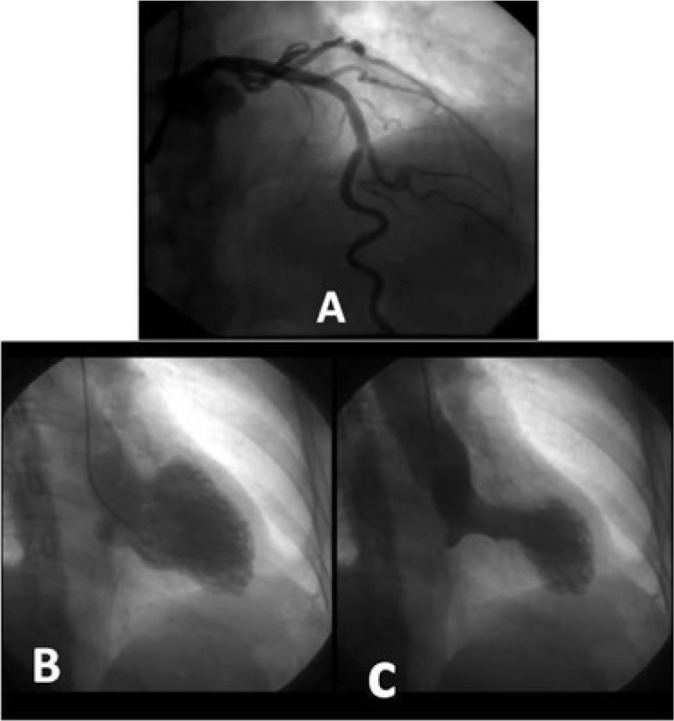
A) Cineangiocorography (CATH) from October 14, 2015 showing no signal of obstructive alterations. B and C) CATH (diastole and systole, respectively) from October 14, 2015 showing left ventricular abnormality, with the typical Takotsubo format.

The patient presented three events of Takotsubo syndrome (TTS) (in 2013, 2015, and 2018). Typical clinical signs of TTS could be seen in all circumstances: she was admitted in the emergency room (ER) with specific precordial pain and short breathing. The electrocardiogram (ECG) showed inverted, symmetric, and peaked T wave (in each event, a different heart wall was stricken: 2013, anterolateral; 2015, anterior; 2018, septal) (Figures 1A and 1B). In the first event, an echocardiogram (echo) showed the left ventricle with segmental alteration, anteroseptal dyskinesia, and apical aneurysmatic dilatation (Figure 2). In the second, it showed tiny ejection fraction for a segmental functional deficit of the anterior wall (Figure 3). And in the third, there was alteration of segmental mobility in the septal wall (Figure 4). None of the events showed significant alterations in cardiac enzymes such as troponin or creatine-phosphokinase-MB (or CPK-MB). In the first two events, at the beginning of precordial pain (first hours), the patient was submitted to an urgent cineangiocorography (CATH) that showed no sign of obstructive alterations (Figure 5A) and the ballooning left ventricular (LV) form, typical of TTS (Figures 5B and 5C).

In all cases, the patient was hospitalized for five days, presenting a full recovery from the symptoms and mobility wall alteration without further complications.

## QUESTIONS

- What is TTS?
- What are the probable causes for this disease?
- What would be the clinical challenges?
- What would be a good technique for the diagnosis of TTS?
- What would be the surgical challenges?

### Discussion of Questions

**Question A.** Takotsubo cardiomyopathy refers to a reversible syndrome characterized by an LV systolic dysfunction with no coronary obstructive cause^[1]^. The singular TTS characteristics are the LV apical ballooning (Takotsubo format), LV akinetic apical region, and hyperkinetic base (compensatory stimulation)^[2-4]^. This syndrome is related to an episode of physical or emotional stress (trigger) that can induce a high peak of catecholamines on the patient’s blood^[5]^.

**Question B.** The most accepted physiopathological theory for TTS proposes that an autonomic response for a high-stress episode induces an overload of cortisol and catecholamines in the patient’s blood. Also, there is a differential response for the peak of epinephrine in the beta-2 adrenergic receptors (negative response to high levels of epinephrine) and G-protein (beta-1) coupled receptors (positive response to high levels of epinephrine), and a differential aggrupation of those different receptors in specific areas of the ventricles (high presence of beta-1 receptors in the apical area with low beta-2, and high beta-2 receptors in a basal area with low beta-1)^[6]^. Those characteristics produce the apical-basal gradient of hypocontractility and hypercontractility, converging in the Takotsubo format of the heart^[7]^. Along with that, there is a vasospasm response of the epicardium and microvascular tissues which contributes to the ischemia and the similar aspect of the disease to myocardial infarction^[5,8]^.

**Question C.** There are no specific clinical signs suggestive of TTS. Most patients are admitted to the ER with typical precordial pain and short breathing, ECG with inverted, symmetric, and peaked T wave in one wall derivations added to ST-segment alterations^[9]^, and echo with a reduced ejection fraction, which are signs that make any doctor suspects of myocardial infarction (acute coronary syndrome [ACS])^[7,10]^. In 2004, scientists proposed the Mayo Clinic diagnostic criteria to help doctors diagnosing TTS. This criteria scores transient LV dysfunction with wall motion abnormalities extended beyond a single epicardial vascular distribution, absence of obstructive coronary disease, ECG with ST-elevation and T-wave inversion, and absence of pheochromocytoma or myocarditis as a base for diagnosing TTS^[11]^.

**Question D.** That is the most problematic issue of this disease, just an urgent CATH, that shows no obstructive alterations, is able to differentiate a myocardial infarction from a TTS, which changes completely the clinical trial.

**Question E.** Analyzing data from the international Takotsubo Registry, it clearly stays that TTS is more frequently caused by a physical than an emotional trigger (36% physical, 27.7% emotional, 7.8% both, and 28% with no triggering event). Added to that, surgery, as physical stress, is one of the most recurrent triggers for high peaks of catecholamines in patients, being TTS a major complicator in the postoperative period of general surgery operations. TTS was already reported in open gastrectomy, in complications of liver transplant, and even in a caesarean section^[1,11-13]^. Afonso et al. showed, in a systematic review, that TTS can be an early complication of cardiac surgery originating from the administration of inotropic drugs, postoperative anxiety, hypoperfusion during cardiopulmonary bypass, and pain^[12]^. It is noteworthy, from the surgical point of view, the description of isolated cases of TTS in the postoperative period of cardiac surgeries, especially those associated with mitral valve disease. This detail cannot fail to be considered in cases that present a picture suggestive of acute myocardial infarction with normal coronary arteries. Suspicion is important because the use of amines is still indicated, and beta-blockers should be used. The suspicion of obstructive hypertrophic cardiomyopathy may lead to the indication of surgical treatment, a fact that is countersense since the hypertrophy of TTS is reversible.

## BRIEF CONSIDERATION OF THE CASE REPORTED

The case reported stands out because of the rarity of > 2 events in one patient. There are just a few descriptions in the bibliography of this characterized case: the three different types of TTS (anterolateral, anterior, and septal) in the same person. Recurrences of this syndrome are infrequently recorded. In the largest series thus far, consisting of 88 patients, a recurrence rate of 2.7% has been reported. In cases of recurrence, one may cautiously speculate that perhaps there is a genetic predisposition towards developing such a reversible syndrome. Of note, Japanese investigators have detected CD36 deficiency in one such patient. This would suggest that certain genetic profiles may be more susceptible to develop stress-induced cardiomyopathy. Recently, Lau et al. (2021) published probably the largest series, including 519 patients with a confirmed diagnosis of TTS^[14]^. The patients were followed for 5.2 years and treatment with beta-blockers were associated with lower risk of recurrence or death. Otherwise, no association was observed between treatment with angiotensin-converting enzyme (ACE) inhibitors or angiotensin-receptor blockers and recurrence or death.

In all the events, the patient was treated with acute thoracic pain protocol (acetylsalicylic acid, coronary vasodilator, and analgesics), with complete reversion of all the symptoms and ECG and echo alterations, with no further complications. Two months after each event, control ECG and echo had been made, presenting no alterations that suggested ischemia, and ejection fraction in 70%, with no alterations of segmental mobility (full recovery and no sequels).

## LEARNING POINTS

- TTS is a disease that is most prevalent in adult women^[4]^.
- TTS occurs in approximately 1-2% of the population with propensity to ACS.
- TTS is of difficult diagnosis because of its similarity to an obstructive ACS.
- ECG (inverted, peaked, and symmetric T wave) and echo (low ejection fraction) are the primary ways to suspect from a TTS and start a further investigation.
- Cineangiocoronariography is the gold standard method for the TTS differential diagnosis.
- Recurrences of this syndrome are rare and infrequently recorded.
- In a series of 519 patients, followed-up for 5.2 years, treatment with beta-blockers was associated with lower risk of recurrence or death. Otherwise, no association was observed between treatment with ACE inhibitors or angiotensin-receptor blockers and recurrence or death.

**Table:** 

Authors’Roles & Responsibilities	
PHRS	Substantial contributions to the conception or design of the work; final approval of the version to be published
JRSJ	Substantial contributions to the conception or design of the work; final approval of the version to be published
PRBE	Substantial contributions to the conception or design of the work; final approval of the version to be published
